# Microsurgical Resection and Posterior Stabilization of a Delayed Solitary Thoracic Vertebral Metastasis From a Malignant Triton Tumor: A Case Report

**DOI:** 10.7759/cureus.108116

**Published:** 2026-05-01

**Authors:** Mars Abdaev, Yuliy Kubetsky, Evgeniy Loparev

**Affiliations:** 1 Neurosurgery, Federal Neurosurgical Center (FSBI), Novosibirsk, RUS

**Keywords:** case report, malignant peripheral nerve sheath tumor, malignant triton tumor, neurosurgical oncology, posterior spinal stabilization, spinal metastasis, thoracic vertebra

## Abstract

Malignant triton tumor (MTT) is an aggressive variant of malignant peripheral nerve sheath tumor (MPNST) characterized by rhabdomyoblastic differentiation and an unfavorable prognosis. Spinal metastases from this tumor are exceptionally uncommon, and management strategies remain unstandardized and largely based on anecdotal evidence. This report presents the case of a 37-year-old woman with a history of retroperitoneal MTT that had been radically resected (R0). The patient developed gradually progressive thoracic back pain without neurological deficit. Magnetic resonance imaging revealed a destructive lesion of the T10 vertebral body consistent with an isolated metastatic focus, with no signs of local recurrence at the primary tumor site. Following a multidisciplinary discussion, surgical treatment was performed without prior biopsy. Microsurgical resection of the vertebral lesion with posterior stabilization from T9 to T11 was successfully completed. Postoperatively, the patient experienced significant pain relief and preserved neurological function. Histopathological examination confirmed metastatic MTT. During follow-up, systemic disease progression was observed; however, local spinal control remained stable. Two years after surgery, there was no evidence of local recurrence or instrumentation failure. This case highlights the potential role of surgical management in selected patients with isolated spinal metastasis from MTT to achieve durable local control and functional preservation.

## Introduction

Malignant triton tumor (MTT) is a rare and highly aggressive subtype of malignant peripheral nerve sheath tumor (MPNST) characterized by rhabdomyoblastic differentiation. MPNSTs account for approximately 5-10% of all soft tissue sarcomas and are associated with aggressive biological behavior and poor prognosis [1,2].

These tumors most commonly arise in association with major peripheral nerves but have been reported in various anatomical locations. Compared with conventional MPNSTs, MTTs are associated with significantly worse clinical outcomes [1-3].

Spinal metastases from MPNSTs are exceptionally uncommon, and isolated vertebral involvement has been reported only sporadically in the literature [3,4]. Consequently, clinical experience regarding optimal management strategies for spinal metastatic disease in this context remains limited and is largely derived from individual case reports. Surgical decision-making in such cases is challenging and must balance oncological control, neurological preservation, and spinal stability.

We present a case of an isolated thoracic vertebral metastasis from a MTT treated with microsurgical resection and posterior stabilization, highlighting the role of surgical management in achieving durable local control and functional preservation in a carefully selected patient.

## Case presentation

**Figure 1:**
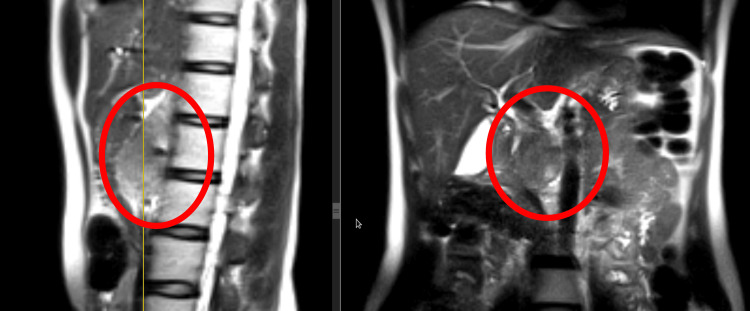
MRI of the chest, sagittal and coronal STIR sequences, demonstrating a heterogeneous posterior mediastinal tumor. STIR: short tau inversion recovery

A 37-year-old female patient initially presented in 2019 with right-sided epigastric pain. Radiological evaluation revealed a large mass located in the posterior mediastinal and retroperitoneal region. Magnetic resonance imaging (MRI) demonstrated a heterogeneous soft tissue tumor in the posterior mediastinum (Figure 1).

**Figure 2:**
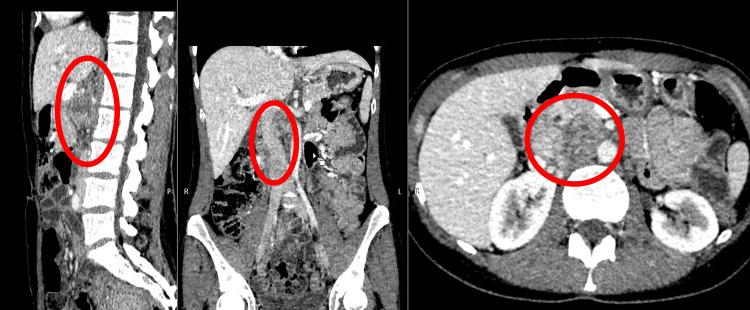
CT angiography of the aorta and its branches (2019), demonstrating the relationship between the tumor and major vascular structures.

To assess the relationship between the tumor and major vascular structures, computed tomography (CT) angiography of the aorta and its branches was performed, revealing close anatomical proximity without evidence of vascular invasion (Figure 2).

Histopathological examination confirmed a MPNST with rhabdomyoblastic differentiation (MTT), FNCLCC (Fédération Nationale des Centres de Lutte Contre le Cancer) grade 2. In August 2019, the patient underwent radical surgical treatment consisting of laparotomy, total resection of a retroperitoneal tumor, resection of the inferior vena cava with linear prosthetic reconstruction, resection of the left renal vein, and arteriolysis of the right renal artery. Postoperative follow-up demonstrated no evidence of local recurrence of the primary tumor.

**Figure 3:**
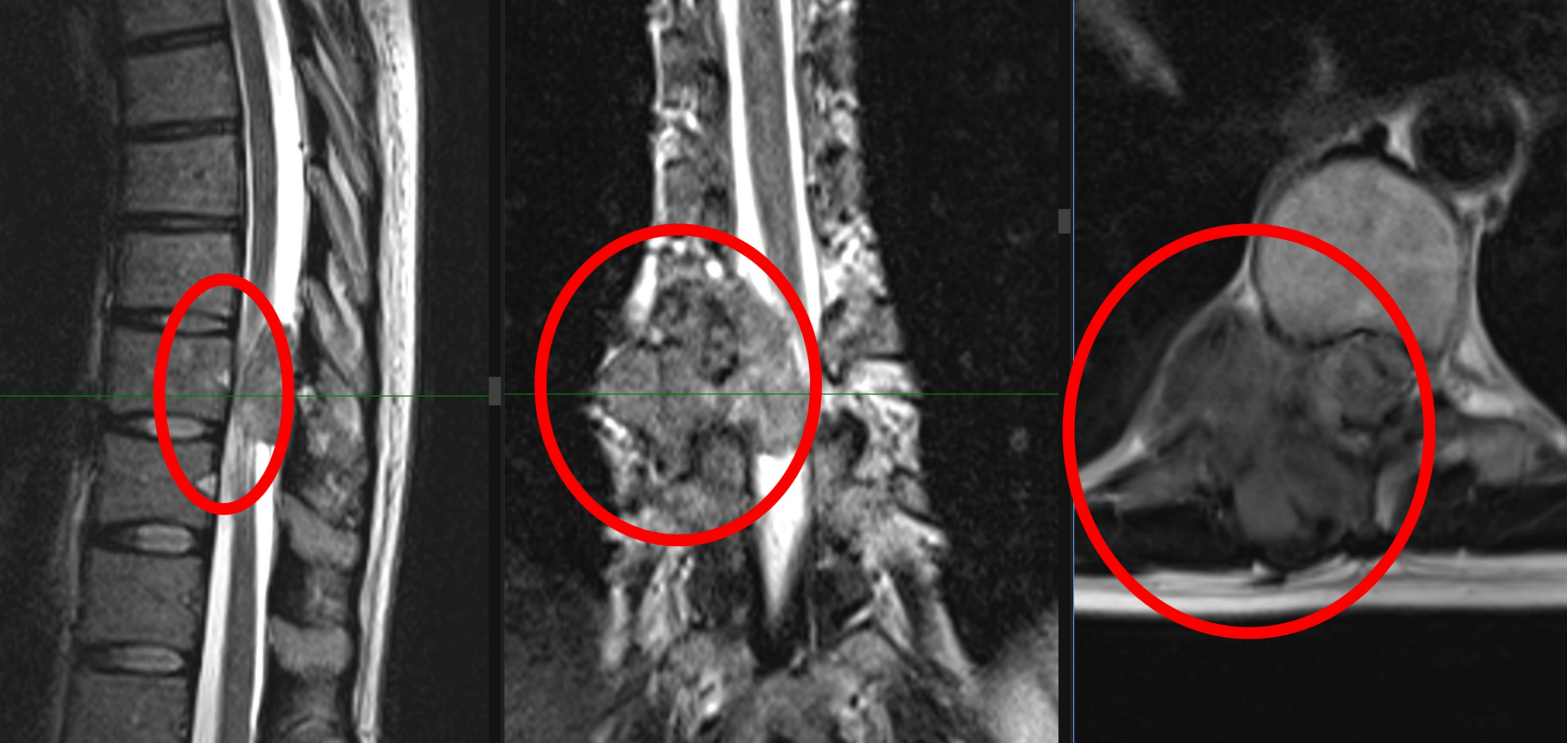
Preoperative MRI of the thoracic spine (2023), including sagittal and coronal T2-weighted sequences and axial T1-weighted post-contrast images, demonstrating a solitary metastatic lesion of the T10 vertebral body.

In September 2022, the patient developed progressively worsening thoracic back pain with radiation to the right scapular and costal regions, without neurological deficit on clinical examination. MRI of the thoracic spine (2023), including sagittal and coronal T2-weighted sequences and axial T1-weighted post-contrast images, demonstrated a solitary destructive lesion of the T10 vertebral body consistent with metastatic involvement (Figure 3). The circled area highlights the lesion. The preoperative spinal instability neoplastic score (SINS) was 9, corresponding to a potentially unstable lesion. This objective assessment, together with severe therapy-resistant pain and destructive involvement of the T10 vertebral body, supported the decision to perform posterior stabilization in addition to tumor resection.

Prior to admission to our institution, the patient underwent comprehensive systemic staging under the supervision of an oncologist at the referring center, including positron emission tomography (PET)-CT, abdominal ultrasonography, and MRI of the neuraxis, all of which demonstrated no evidence of additional metastatic disease. The patient was referred to our center with findings consistent with an isolated vertebral lesion.

Prior to surgical intervention, the case was reviewed by a multidisciplinary tumor board, including neurosurgeons and oncologists. Alternative diagnoses, including primary vertebral tumor, lymphoma, and infectious processes, were considered during multidisciplinary evaluation. However, the patient’s known history of MTT, radiologically consistent solitary lesion, and absence of systemic disease on comprehensive staging, including PET-CT, strongly supported the diagnosis of metastatic disease. Given the patient’s oncological history, isolated vertebral involvement, absence of local recurrence of the primary tumor, and lack of systemic disease progression at that time, surgical treatment was recommended. Preoperative biopsy was not performed, as imaging findings and clinical context were consistent with metastatic disease, and histological verification was unlikely to alter the treatment strategy while potentially delaying definitive treatment.

In March 2023, the patient underwent single-stage microsurgical resection of the T10 vertebral lesion with posterior spinal stabilization using the XIA III system from Th9 to Th11. The procedure was not a total spondylectomy. The surgery was performed under general endotracheal anesthesia with the patient in the prone position. A posterior midline approach was used, with exposure of the posterior elements from T7 to L1.

During mobilization of the right costotransverse region, a tumor with relatively well-defined borders from the surrounding muscles was identified. Bilateral polyaxial pedicle screws were inserted into T9 and T11 using O-arm navigation, and intraoperative imaging confirmed correct screw positioning.

Bone resection along the tumor margin was performed using an ultrasonic bone scalpel. The tumor was carefully mobilized, separated from the dural sac and parietal pleura, and removed in two fragments within macroscopically healthy tissue planes. Although formal histological margin assessment was not available, no residual tumor was identified intraoperatively. Careful mobilization, removal within macroscopically intact tissue planes, and meticulous hemostasis were used to minimize the risk of local tumor dissemination.

**Figure 4:**
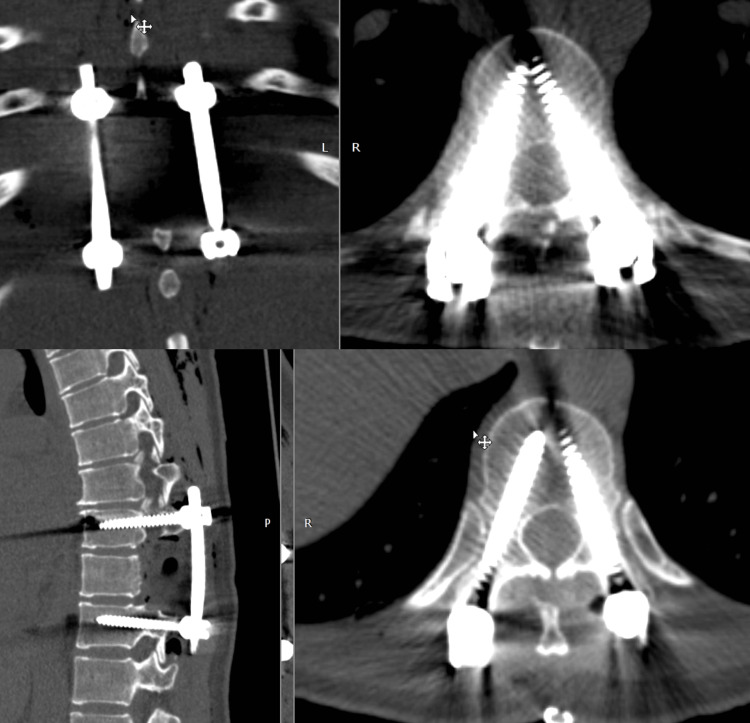
Postoperative CT of the thoracic spine demonstrating resection of the T10 lesion and posterior spinal stabilization.

The tumor tissue, together with bone fragments, was sent for histopathological examination. Rods were mounted, and the posterior stabilization system was assembled. Intraoperative neurophysiological monitoring, including motor evoked potentials and electromyographic control, remained stable throughout the procedure. Estimated blood loss was 200 mL. Postoperative CT confirmed adequate decompression and correct positioning of the instrumentation (Figure 4). The postoperative course was uneventful, with significant pain relief and preservation of neurological function.

The lesion was removed in two fragments within macroscopically healthy tissue planes along the borders of the dura mater and parietal pleura. Although formal histological margin assessment was not available, no residual tumor was identified intraoperatively, and postoperative imaging showed adequate decompression and satisfactory implant positioning.

**Figure 5:**
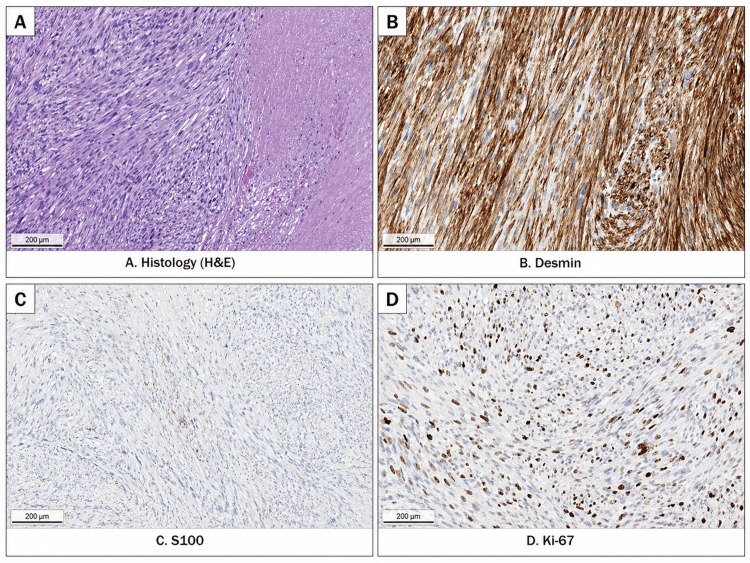
Histopathological and immunohistochemical findings of the metastatic malignant triton tumor. (A) Hematoxylin and eosin staining demonstrating spindle-cell tumor proliferation with nuclear pleomorphism, necrosis, and mitotic activity. (B) Desmin immunostaining showing diffuse positive expression, supporting rhabdomyoblastic differentiation. (C) S100 immunostaining showing focal positive expression. (D) Ki-67 immunostaining demonstrating an increased proliferative index of approximately 33%.

Histopathological examination confirmed metastatic MPNST with rhabdomyoblastic differentiation, consistent with MTT. Microscopically, the tumor consisted of spindle cells with marked nuclear pleomorphism arranged in densely cellular intersecting fascicles, with areas of necrosis and mitotic activity (up to four mitoses per 10 high-power fields). Immunohistochemical analysis demonstrated diffuse desmin positivity, focal S100 expression, focal loss of H3K27me3 expression in a subset of tumor cells, and negative staining for SRY-related HMG-box 10 (SOX10) and glial fibrillary acidic protein (GFAP). The proliferative index (Ki-67) was approximately 33%. The tumor was classified as FNCLCC grade 2. Representative histopathological and immunohistochemical findings are shown in Figure 5.

**Figure 6:**
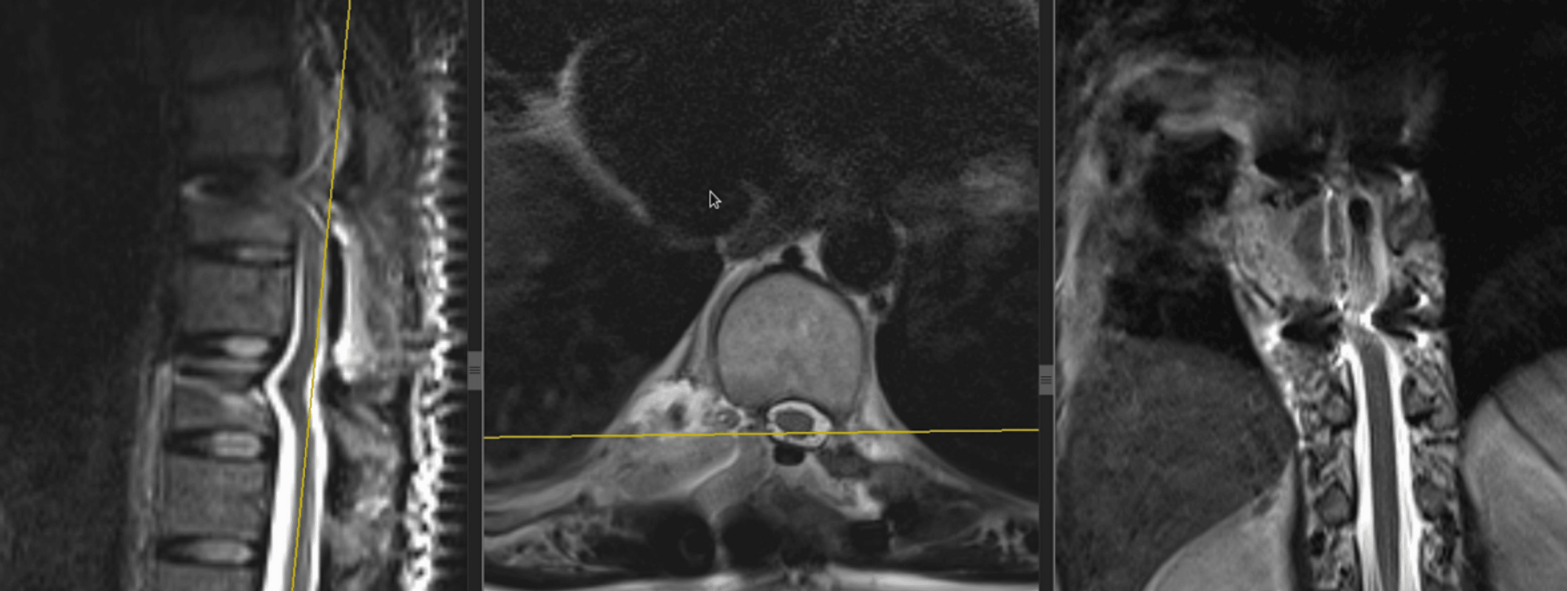
Follow-up MRI of the thoracic spine two years after surgery, including sagittal, axial, and coronal T2-weighted sequences, showing no evidence of local tumor recurrence.

Follow-up MRI of the thoracic spine performed two years after surgery, including sagittal, axial, and coronal T2-weighted sequences, demonstrated no evidence of local tumor recurrence at the operated level (Figure 6). During follow-up, systemic disease progression with pulmonary and soft tissue metastases was documented; however, the operated spinal segment remained locally controlled, and the patient remained neurologically intact with satisfactory self-reported quality of life at the latest follow-up.

During the subsequent 12 months, the patient received adjuvant oncological treatment, including 12 sessions of radiotherapy and seven cycles of systemic chemotherapy. At the latest follow-up, she reported no complaints related to the operated spinal level, remained neurologically intact, and had a satisfactory overall clinical condition.

## Discussion

MTTs are associated with a particularly poor prognosis, with reported five-year survival rates ranging from approximately 5% to 15% in most series [1-3]. Spinal involvement in MTT is extremely rare, and available evidence regarding optimal management is limited to isolated case reports and small series [3,4]. In most published cases, spinal disease occurs in the setting of widespread systemic progression, which often limits the role of aggressive local treatment [4-6].

In contrast, the present case is notable for the development of a solitary thoracic vertebral metastasis more than three years after radical resection of the primary retroperitoneal tumor, with no evidence of local recurrence or disseminated disease at the time of spinal involvement. This timing distinguished the case from terminal disseminated disease and created a window of opportunity for early aggressive local intervention aimed at symptom control and durable local disease management. Thus, the timing of spinal metastasis relative to systemic progression may be an important factor in surgical decision-making, supporting a proactive local-control strategy rather than a purely palliative approach in carefully selected patients.

The decision to proceed with surgery without preoperative biopsy was based on the patient’s known oncological history, characteristic imaging findings, and the fact that histological confirmation would not have altered the treatment strategy. Similar approaches have been described in selected cases of solitary spinal metastases, where prompt surgical management is prioritized to prevent neurological deterioration and alleviate severe pain [5-7].

Microsurgical resection of the affected vertebral segment combined with posterior spinal stabilization resulted in significant pain relief and preservation of neurological function. These outcomes are consistent with previously reported benefits of surgical decompression and stabilization in patients with isolated spinal metastases, even in the context of aggressive underlying malignancies [5-7].

Although the patient subsequently developed systemic disease progression, including pulmonary and soft tissue metastases, the absence of local recurrence at the surgical site over a two-year follow-up period is particularly noteworthy given the aggressive nature of MTT. Although the overall treatment strategy remained palliative, surgical intervention played a significant role in preserving neurological function, maintaining structural stability, and supporting quality of life. In carefully selected patients, long-term preservation of neurological function should therefore be considered a relevant surgical endpoint, even when systemic cure is not achievable.

Overall, this case underscores the importance of individualized, multidisciplinary decision-making in rare oncological scenarios and suggests that radical surgical management may provide meaningful local control and symptomatic benefit in carefully selected patients with isolated vertebral metastasis from MTT.

## Conclusions

MTT is an aggressive neoplasm with a high risk of systemic progression and limited published experience regarding spinal metastatic involvement. In selected patients with delayed solitary vertebral metastasis and controlled primary disease, surgical treatment may provide meaningful local control, preservation of neurological function, and maintenance of quality of life.

Although the overall treatment strategy remains palliative, this case supports the role of individualized multidisciplinary decision-making and targeted spinal stabilization as a valuable surgical strategy when systemic cure is not achievable. Given that this is a report of a single case, these findings should be interpreted cautiously and viewed as hypothesis-generating rather than definitive evidence for broader treatment recommendations.
